# Human RECQ1 Interacts with Ku70/80 and Modulates DNA End-Joining of Double-Strand Breaks

**DOI:** 10.1371/journal.pone.0062481

**Published:** 2013-05-01

**Authors:** Swetha Parvathaneni, Alexei Stortchevoi, Joshua A. Sommers, Robert M. Brosh, Sudha Sharma

**Affiliations:** 1 Department of Biochemistry and Molecular Biology, College of Medicine, Howard University, Washington, DC, United States of America; 2 Laboratory of Molecular Gerontology, National Institute on Aging, National Institutes of Health, Baltimore, Maryland, United States of America; University Medical Center Hamburg-Eppendorf, Germany

## Abstract

Genomic instability is a known precursor to cancer and aging. The RecQ helicases are a highly conserved family of DNA-unwinding enzymes that play key roles in maintaining genome stability in all living organisms. Human RecQ homologs include RECQ1, BLM, WRN, RECQ4, and RECQ5β, three of which have been linked to diseases with elevated risk of cancer and growth defects (Bloom Syndrome and Rothmund-Thomson Syndrome) or premature aging (Werner Syndrome). RECQ1, the first RecQ helicase discovered and the most abundant in human cells, is the least well understood of the five human RecQ homologs. We have previously described that knockout of RECQ1 in mice or knockdown of its expression in human cells results in elevated frequency of spontaneous sister chromatid exchanges, chromosomal instability, increased load of DNA damage and heightened sensitivity to ionizing radiation. We have now obtained evidence implicating RECQ1 in the nonhomologous end-joining pathway of DNA double-strand break repair. We show that RECQ1 interacts directly with the Ku70/80 subunit of the DNA-PK complex, and depletion of RECQ1 results in reduced end-joining in cell free extracts. In vitro, RECQ1 binds and unwinds the Ku70/80-bound partial duplex DNA substrate efficiently. Linear DNA is co-bound by RECQ1 and Ku70/80, and DNA binding by Ku70/80 is modulated by RECQ1. Collectively, these results provide the first evidence for an interaction of RECQ1 with Ku70/80 and a role of the human RecQ helicase in double-strand break repair through nonhomologous end-joining.

## Introduction

A DNA double-strand break (DSB) is particularly detrimental to genome integrity [1]. DSBs are generated naturally in cells during programmed genome rearrangements [2], [3], and as a consequence of problems in DNA metabolism, such as replication fork collapse or DNA damage induced by extrinsic mutagens including radiations [4]. Unrepaired DSBs lead to loss of genetic information and mutagenesis or cell death [5], [6]. Therefore, accurate repair of DSBs is indispensable to genome homeostasis and cell survival.

Homologous recombination (HR) and nonhomologous end-joining (NHEJ) are mechanistically distinct DNA repair pathways that contribute substantially to DSB repair in mammalian cells [7]. HR utilizes an unbroken, homologous sequence as a template for repair of a DSB, thereby ensuring that any genetic information disrupted or lost at the site of the break is regained accurately. DSB repair by HR is mediated by members of the conserved Rad52 epistasis group and several other less conserved accessory factors [4], [8]. NHEJ is a second prominent pathway for DSB repair in which broken ends are healed without the requirement for significant sequence homology [9]. NHEJ is therefore a less accurate repair mechanism and may result in the loss or gain of nucleotides at the break point. The core NHEJ machinery includes the end-binding heterodimeric proteins Ku70/Ku80, the DNA-PKcs protein kinase, and the complex consisting of DNA ligase IV, XRCC4 and XLF [9]. Mammalian cells preferentially utilize NHEJ for DSB repair throughout the cell cycle and exclusively during G1 to early S phase when the homologous template is unavailable for HR [10]. A competition may exist between NHEJ and HR [11], and inhibition of the HR pathway by components of NHEJ has been reported [12], [13]. A number of proteins including the MRE11/RAD50/NBS1 (MRN) complex, BRCA1 and PARP-1 are shown to modulate both pathways but it is yet unclear how the choice is made between the HR and NHEJ pathways for the repair of a DSB [14].

RecQ helicases contribute diverse activities towards genome maintenance in response to a variety of DNA lesions [15], [16]. RECQ1 protein is the smallest of the five human RecQ homologs, and shares maximum homology to the prototype E. coli RecQ. RECQ1 helicase binds and preferentially unwinds model structural intermediates of DNA repair, such as forked duplexes, D-loops, and Holliday junctions [17], [18]. Besides conventional unwinding, RECQ1, like BLM and WRN, also promotes the branch migration of recombination intermediates such as Holliday junctions and D-loops in an ATP-dependent fashion [17], [19]. Compared with their helicase activity which is limited in its processivity to 25–100 base pairs (bp), the branch migration by RecQ helicases is more processive and can occur over several kilobases [19], [20]. RECQ1 specifically catalyzes unidirectional branch migration, which may be instrumental in specific disruption of toxic, nonproductive intermediates of HR during DSB repair *in vivo* [19]. Remarkably, RECQ1 and other human RecQ proteins have an intrinsic ability to promote annealing of complementary single strand DNA in an ATP-independent manner [17]. Due to their diverse biochemical activities, RecQ proteins are potentially critical players in the DSB repair pathways [21].

Functions of RecQ helicases are critical for genome maintenance [15]. Germline mutations in the human RecQ helicase homologs WRN, BLM and RECQ4 are responsible for the rare genetic disorders of Werner Syndrome, Bloom Syndrome, and Rothmund-Thomson Syndrome, respectively, all of which are characterized by chromosomal instability and predisposition to cancer. A human disease has not yet been linked to defects in RECQ1 or RECQ5, and the evidence for their roles in the processes of DNA repair is only beginning to emerge. Several lines of evidence suggest altered DSB repair in RECQ1-deficient cells [22], [23]. Knockout (KO) of the RECQ1 gene in mice [24] or suppression of its expression in human cells [25] results in increased sensitivity to ionizing radiation, chromosomal instability and elevated sister chromatid exchanges (SCEs) that represent reciprocal DNA exchanges between homologous chromosomes. Consistent with a defect in DNA strand break repair, RECQ1-deficient cells accumulate potentially unresolved recombination events as displayed by Rad51 foci and exhibit constitutively increased DSBs [24], [25]. We have recently determined that depletion of RECQ1 does not significantly influence the DSB-induced HR frequencies as determined by homology-directed repair of I-SceI-induced DSBs in human cells containing an integrated DR-GFP reporter substrate [26]. It remains possible, though, that RECQ1 contributes more directly to another pathway of DSB repair, such as NHEJ.

This study reports a physical and functional interaction of RECQ1 with the Ku70/80 subunits of the DNA-PK complex. Results presented here provide the first evidence for a direct role of RECQ1 in DSB repair by NHEJ.

## Materials and Methods

### Recombinant Proteins

Recombinant human RECQ1 helicase was purified as described previously [17]. Purified recombinant Ku70/80 was generously provided by Dr. Dale Ramsden (University of North Carolina at Chapel Hill) [27].

### Cell Lines, Culture Conditions and DNA Damage Treatment

Human HeLa (ATCC), human glioma isogenic MO50J cells that lack DNA-PK activity due to a frameshift mutation in DNA-PKcs and reconstituted MO59K cells [28] as well as the embryonic fibroblasts from wild type (WT) and RECQ1 knockout (KO) mice [24] were grown in Dulbecco’s modified Eagle’s medium (DMEM) (Invitrogen) supplemented with 10% fetal bovine serum (Hyclone Laboratories), 100 U/ml penicillin and 100 µg/ml streptomycin (Invitrogen). Cells were grown in a humidified 5% CO_2_ incubator at 37°C. To induce DNA breaks, exponentially growing cells were treated with the radiomimetic drug neocarzinostatin (NCS; Sigma) and allowed to recover at 37°C for indicated time. Stock solution of the DNA-PKcs inhibitor Nu7026 (Sigma) was made in DMSO and the same volume of DMSO was added to the control plates.

### Immunoprecipitation

HeLa nuclear extract was prepared as previously described [29]. Extracts were incubated with Protein G-Dynal beads coupled with polyclonal antibody against human RECQ1 (Bethyl Laboratories), mouse monoclonal against Ku70/80 (Abcam), normal rabbit IgG or normal mouse IgG (Vector Labs) at 4°C for 90 min, and the immunecomplexes were eluted with 2x SDS-sample buffer following three washes with lysis buffer. Where indicated, nuclear extract was pre-incubated with benzonase (Sigma, 50 U/ml, 2 h at 4°C) or ethidium bromide (EtBr) (50 µg/ml) was present during the immunoprecipitation incubation. Proteins were resolved by 8–16% SDS-PAGE, transferred onto PVDF membrane and subjected to Western detection of RECQ1 (1∶750, Santa Cruz Biotech), Ku70 and Ku80 (both 1∶500, Santa Cruz Biotech).

### GST Pull-down

Wild-type and truncated fragments of GST-RECQ1 fusion proteins were overexpressed in BL21(DE3) pLysS by 1 mM isopropyl-B-d-thiogalactopyranoside induction for 8 h at 23°C. The bacterial cell pellet was sonicated in lysis buffer (phosphate-buffered saline (PBS), 10% glycerol and 0.4% Triton X-100) and the lysate was clarified by centrifugation at 35,000 rpm for 1 h at 4°C. Approximately 1 ml of the resulting supernatant was incubated with 100 µl of glutathione *S*-transferase beads (50% v/v) for 1 h at 4°C. The beads were washed three times with 1 ml lysis buffer, and split into two aliquots, one for binding experiments and one for determination of expression by Coomassie Blue staining. For binding experiments, protein-bound beads were incubated overnight at 4°C with 500 µg of lysate from untreated HeLa cells, or with 1 mg of bacterial extracts expressing recombinant his-tagged Ku70, Ku80 or N-Ku80. The beads were subsequently washed five times with 1 ml of lysis buffer and eluted by boiling with 2x SDS-sample buffer. Eluted proteins were electrophoresed on 8–16% polyacrylamide SDS gels and either stained with Coomassie Blue to demonstrate protein loading or transferred onto PVDF membranes for Western detection of Ku70/80 bound to GST-RECQ1 proteins using anti-Ku70 or anti-Ku80 antibody.

### ELISA for RECQ1-Ku70/80 Interaction

Either BSA or purified recombinant human Ku70/80 (12.5 nM) was coated onto microtiter plates. Following blocking with 3% BSA, appropriate wells were incubated with the indicated concentrations of purified recombinant human RECQ1 (0–100 nM). DNaseI (Sigma, 100 U/ml) or EtBr (50 µg/ml) was included in the incubation with RECQ1 in the binding step in the corresponding wells to test for DNA-mediated protein interaction. Following washing, Ku70/80-bound RECQ1 was detected by ELISA using rabbit polyclonal antibody against RECQ1. The values represent the mean of three independent experiments performed in duplicate with standard deviation (SD) indicated by error bars.

### Immunofluorescence Assay for Colocalization

Cells grown on glass coverslips to about 70% confluence were untreated or treated with NCS for 3 h, fixed with 3.7% paraformaldehyde for 10 min, and permeabilized in 0.5% Triton X-100 solution for 10 min at room temperature. Cells were blocked with 10% fetal calf serum in PBS and incubated with rabbit polyclonal anti-RECQ1 antibody (1∶500, Santa Cruz Biotech) and/or mouse monoclonal Ku70/80 antibody (1∶200, Abcam) overnight at 4°C in a humid chamber. After washes in PBS with 0.1% Tween-20, cells were incubated with Alexa Fluor 488 goat anti-rabbit IgG (1∶400; Invitrogen) and Alexa Fluor 568 goat anti-mouse IgG (1∶400, Invitrogen) secondary antibodies for 1 h at 37°C. Cells were washed four times with PBS containing 0.1% Tween-20, mounted with Prolong Gold containing DAPI (Invitrogen), and analyzed by confocal microscopy (Olympus).

### siRNA Transfection

Depletion of RECQ1 or Ku80 was achieved by transfecting HeLa cells with a scrambled control, RECQ1 or Ku80 siRNA (siGenome smartpool, Dharmacon) at a final concentration of 10 nM using Lipofectamine 2000 transfection reagent as per the manufacturer’s instructions (Invitrogen). Following transfection, cells were cultured in regular growth medium for 36 h before harvesting for biochemical fractionation or lysate preparation with or without NCS treatment.

### Biochemical Fractionation

HeLa cells grown at 70–80% confluence were mock-treated or treated with indicated concentration of NCS for 3 h. Cells were harvested in cold PBS and total cell lysate was made using RIPA buffer. For subcellular fractionation, equivalent cell pellets were resuspended in 2 packed cell volumes of buffer containing 20 mM Tris-HCl (pH 7.4), 2.5 mM MgCl_2_, 0.5% Nonidet P-40, 1 mM phenylmethylsulfonyl fluoride (PMSF), 1 mM dithiothreitol (DTT) and protease inhibitors (Roche), and incubated on ice for 10 min. Following centrifugation at 10,000 rpm for 2 min, the supernatant was transferred to a new Eppendorf tube and designated the “soluble” fraction containing cytoplasmic proteins. The remaining nuclear pellet was similarly extracted with 2 packed cell volumes of buffer containing 20 mM Tris-HCl (pH 8.0), 0.5 M KCl, 1 mM EDTA, 0.75% Triton-X100, 10% glycerol, 5 mM MgCl_2_, 1 mM PMSF, 1 mM DTT, and protease inhibitors, and the supernatant obtained was designated the “insoluble” fraction containing chromatin bound proteins. Proteins from each fraction were analyzed by Western blotting. ImageJ was used for quantification of Western signal.

### Assay of Ku70/80 (Ku)-DNA Binding Activity

DNA binding activity in cell extracts was analyzed by using a Ku70/Ku86 DNA Repair Kit (Active Motif) according to the manufacturer’s instructions. Briefly, cells were washed, resuspended in hypotonic buffer and the nuclear extract was prepared as directed. To assay for Ku activity, 5 µg of nuclear extract was added in triplicates to the oligonucleotide-coated 96-well plate and incubated for 1 h at room temperature. Monoclonal Ku70/80 antibody was added to the wells and incubated for another 1 h. After washing, wells were incubated with HRP-conjugated secondary antibody for 30 min and colorimetric detection of Ku-DNA binding activity was performed at 450 nm. Each experiment was repeated three times, and data represent the mean of three separate determinations with SD indicated by error bars.

### DNA Substrates for Various Assays

PAGE-purified oligonucleotides used for preparation of DNA substrates were purchased from Midland Certified Reagent Co. 5′-^32^P-Labeled fork duplex DNA substrates used for DNA binding and helicase assays were prepared as described previously [17]. For preparation of EMSA template, 10 µg of the supercoiled pUC19 plasmid vector (2686 bp, Invitrogen) were digested with PvuII-HF endonuclease (NEB) at 37°C for 2 h, the resulting 322 bp and 2364 bp blunt-ended DNA fragments were resolved in 1% TAE agarose and the 322 bp DNA fragment subsequently purified using QIAquick Gel Extraction kit (Qiagen). Gel shift reactions utilized 30 ng of the 322 bp DNA fragment. T4 ligase and NHEJ reactions utilized 100 ng of EcoRI (5′-overhang), SacI (3′-overhang) or PvuII (blunt ends)-linearized pUC19 plasmid DNA.

### Fork Duplex Binding and Unwinding Assays

Reaction mixtures (20 µl) contained 20 mM Tris-HCl (pH 7.5), 10 mM KCl, 8 mM DTT, 5 mM MgCl_2_, 5 mM ATP or ATPγS, 10% glycerol, 80 µg/ml BSA, 0.5 nM fork DNA substrate, and the indicated concentrations of RECQ1 and/or Ku70/80. Reactions were incubated for 15 min at 37°C, followed by addition 20 µl of stop buffer (35 mM EDTA, 0.6% SDS, 25% glycerol, 0.04% bromphenol blue, and 0.04% xylene cyanol) with a 10-fold molar excess of unlabeled competitor oligonucleotide, and samples were loaded onto native 5% (for binding) or 12% (for unwinding) polyacrylamide gels (19∶1 cross-linking ratio) and electrophoresed at 200 V for 2.5 h at 4°C using 1x TBE as the running buffer. The resolved radiolabeled species were visualized with a PhosphorImager and analyzed using ImageQuant software.

### Electrophoretic Mobility Shift Assay (EMSA) with Linear DNA

Binding reactions (20 µl) were performed by incubating purified recombinant RECQ1 and/or Ku70/80 proteins (1.5–100 nM) and DNA probe (30 ng) in 1x EMSA buffer (25 mM Tris HCl (pH 7.5), 150 mM KCl, 100 µg/ml BSA, 5% glycerol, 0.1% Triton X100 and 2 mM DTT) for 15 min at 25°C, followed by electrophoresis in a 6% native polyacrylamide gel in 1x TBE buffer at 150 V for 3.5 h. The gel was stained with SYBR Gold (Life Technologies) and image documented on a UV light box.

### Pull-down of Biotinylated DNA

Biotinylated dsDNA probe was generated by PCR with Biotin-CTGGCGAAAGGGGGATGTGCTGC and CTGGCACGACAGGTTTCCCG primers, using pUC19 plasmid vector as template. The resulting PCR product mimicked the 322 bp PvuII excision fragment of pUC19 and had a single biotin molecule covalently attached to C-nucleotide at the 5′-end of one DNA strand. M-280 Streptavidin Dynabeads (Invitrogen) were used for pull-downs. RECQ1 (25, 50 or 80 nM) and Ku70/80 (12.5 or 160 nM) were either combined immediately prior to incubating with the DNA for 25 min, or one protein was pre-incubated with DNA for 15 min and then another was added and incubation continued for additional 10 min. The biotinylated DNA (60 ng) was mixed with proteins in 40 µl of 1x EMSA buffer and incubated at room temperature (25 min), followed by addition of 20 µL of pre-washed Dynabeads. The beads were incubated with DNA-protein binding mixture for 15 min at room temperature, supernatant was discarded, and the beads resuspended in 0.2 ml of washing buffer (1x EMSA buffer minus glycerol) and the suspension was split in halves for DNA and protein analyses following three washes. Proteins were eluted from the beads in 20 µl of 2x SDS-sample buffer at 90°C for 5 min and analyzed by Western blotting with anti-RECQ1 and anti-Ku80 antibodies. DNA was eluted in 20 µl of 10 mM EDTA (pH 8.2) with 95% formamide at 90°C for 1 min, run on 2% TAE agarose gel and stained with SYBR Gold.

### T4 Ligase Assay

Substrate DNA (100 ng) was mixed in 10 µl of 1x T4 ligase buffer supplemented with 0.2 mg/ml BSA, with indicated amounts of RECQ1 or Ku70/80 protein, pre-incubated for 10 min at room temperature followed by addition of T4 ligase (200 U, NEB) in 10 µl 1x T4 ligase/BSA buffer. The ligation reaction was allowed to continue at room temperature for 2 or 4 h for 5′-cohesive and blunt-ended linear DNA, respectively. Ligation products were recovered by phenol/chloroform extraction, separated in 0.7% TAE agarose gel, visualized by staining with SYBR Gold and represented as an inverted image.

### NHEJ Assay

Cell free extracts were prepared from HeLa or mouse embryonic fibroblasts (MEFs) following standard protocol [30]. Linearized plasmid DNA (100 ng) was incubated with the cell free extracts (0–15 µg) in 50 µl of 1x NHEJ buffer (20 mM Tris Acetate (pH 7.5), 5 mM magnesium acetate, 80 mM potassium acetate, 4 mM ATP, 0.2 mg/ml BSA and 2 mM DTT) for 2 h at room temperature. For antibody interference experiments, up to 3 µg of a specific antibody as indicated or IgG control was included in the reaction mixtures. In immunodepletion experiments, the cell free extracts were pre-incubated with the antibody-bound magnetic beads in 1x NHEJ buffer for 30 min at 4°C, beads were removed on the magnetic platform and the immunodepleted extract was used for NHEJ reaction as described. Upon completion of incubation, the reactions were treated with RNaseA (100 µg/ml), deproteinised with proteinase K, and the end-joined DNA products were extracted with phenol/chloroform, ethanol precipitated in presence of GlycoBlue (Ambion), separated by 0.7% TAE agarose gel, visualized by staining with SYBR Gold and represented as an inverted image.

## Results

Our previous observations that RECQ1 deficiency leads to cellular sensitivity to ionizing radiation or hydrogen peroxide [24], [25], [26] that potentially leads to DSBs raised the possibility that RECQ1 plays a direct role in DSB repair. Although RECQ1 deficiency in human cells did not affect homology-directed repair of I-SceI-induced DSBs [26], a role of RECQ1 in NHEJ had not been addressed previously. Mass spectrometry analyses of RECQ1-immunoprecipitate from HeLa cells that identified PARP-1 as an interacting protein also revealed Ku70/80 heterodimer as a novel RECQ1-partner (data not shown) [26]. Given the critical importance of the Ku70/80 heterodimer in NHEJ repair of DSBs, we characterized the putative interaction of RECQ1 with the Ku proteins.

### RECQ1 Exists in Complex with Ku70/80 Heterodimer

**Figure 1:**
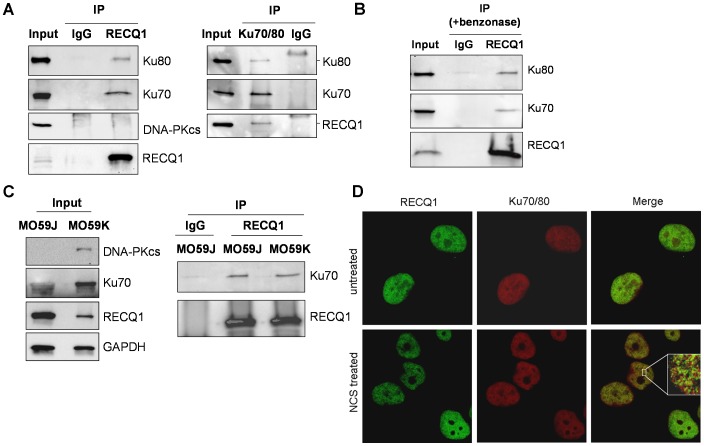
RECQ1 interacts with Ku70/80 *in vivo*. *A*. Co-immunoprecipitation analysis of RECQ1 interaction with Ku70/80 using HeLa nuclear extracts. Immunoprecipitations (IP) with antibodies specific for RECQ1, Ku70/80 and preimmune IgG are indicated. Eluted proteins in immunoprecipitate were analyzed by Western blotting and are indicated. RECQ1 IP contained Ku70 and Ku80 subunits but DNA-PKcs was not detected. Reciprocal co-IPs of Ku70/80 also contained RECQ1. *B.* Association of RECQ1 and Ku70/80 is not mediated via DNA. RECQ1 antibody co-precipitated RECQ1 and Ku70/80 using benzonase-treated extract in IP reaction. *C.* RECQ1 interacts with Ku in DNA-PKcs deficient and proficient cells. Lysates of MO59J (DNA-PKcs deficient) or MO59K (DNA-PKcs proficient) cells were used for IP using RECQ1 antibody or IgG and analyzed by Western blotting as indicated. *D*. Immunofluorescence staining of endogenous RECQ1 and Ku70/80. HeLa cells grown on coverslips were either mock-treated or treated with NCS (100 ng/ml, 3 h). Cells were fixed and immunostained using a mouse monoclonal Ku70/80 antibody (1∶200) and a rabbit polyclonal RECQ1 antibody (1∶500). RECQ1 and Ku70/80 were visualized with Alexa Fluor 488- or Alexa Fluor 568-conjugated secondary antibodies, respectively, followed by confocal microscopy. Inset shows enlarged portion of the nucleus after NCS treatment; colocalization of RECQ1 (green) and Ku70/80 (red) in cells appears yellow in merged images. In all experiments, input corresponds to 5% of total protein used in IP reactions.

To determine if RECQ1 interacts with Ku70/80, we performed reciprocal immunoprecipitation using specific antibodies from HeLa cell nuclear extracts (Fig. 1A). Western blot analyses showed RECQ1 antibody specifically co-precipitated Ku70 and Ku80 and immunoprecipitation of Ku70/80 resulted in co-precipitation of RECQ1 (Fig. 1A). Similar immunoprecipitation using normal IgG failed to pull down RECQ1, Ku70 or Ku80 (Fig. 1A). The presence of EtBr (data not shown) or the use of benzonase-treated extract in immunoprecipitation reaction did not abolish co-precipitation of Ku70/80 and RECQ1, suggesting that the interaction is not mediated by DNA (Fig. 1B).

The Ku complex participates in NHEJ by binding to DSBs and recruiting the catalytic subunit of DNA-PK (DNA-PKcs), thereby forming the active holoenzyme complex [9]. We did not detect DNA-PKcs in RECQ1-immunoprecipitate despite its presence in the input nuclear extract (Fig. 1A). To determine whether the RECQ1-Ku interaction is mediated by DNA-PKcs, we performed immunoprecipitation using extracts prepared from the DNA-PKcs negative (MO59J) and corrected (MO59K) cells. Using GAPDH signal as protein loading control, comparable amounts of RECQ1, Ku70 or Ku80 proteins were present in the nuclear extracts of MO59J and MO59K cells, and RECQ1 antibody co-immunoprecipitated RECQ1 and Ku70 (Fig. 1C). This suggests that the interaction of RECQ1 and Ku complex is independent of DNA-PKcs.

To determine whether RECQ1 and Ku70/80 cooperate after DNA damage, we next examined localization of RECQ1 and Ku70/80 in HeLa cells that were untreated or treated with the DSB inducing agent NCS. RECQ1 and Ku70/80 proteins were detected in HeLa cell nuclei as reported previously [25], [31], and partially colocalized in a punctate pattern following NCS treatment (Fig. 1D). Similar localization pattern for RECQ1 and Ku70/80 were obtained in DNA-PKcs deficient MO59J cells (Fig. S1).

### RECQ1 Interacts with Ku70 and Ku80 *in vitro*

**Figure 2:**
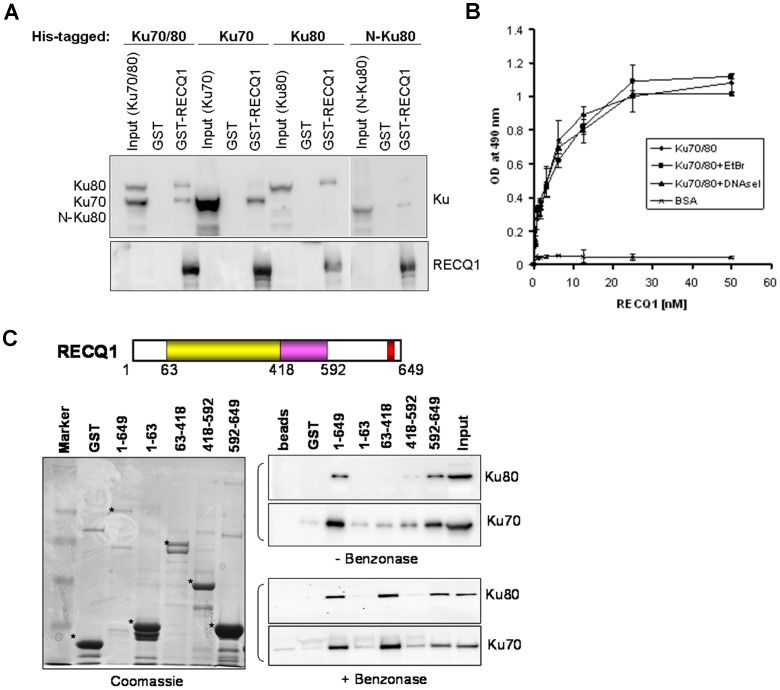
A direct physical interaction between RECQ1 and Ku70/80 *in vitro*. *A.* RECQ1 directly interacts with Ku70 and Ku80. GST or GST fused-full length RECQ1 was incubated with bacterially expressed Ku70/80, Ku70, Ku80 or N-Ku80 (lacking the C-terminus amino acid residues 565–732) followed by extensive washing of the beads, SDS-PAGE, and Western transfer. The blots were probed separately with anti-His (for Ku detection) and anti-RECQ1 antibodies. Input lanes account for 10% of the bacterial lysate expressing Ku protein used in the pull-down reactions. *B.* Recombinant RECQ1 and Ku70/80 proteins interact directly as shown by ELISA. Either BSA or purified recombinant Ku70/80 was coated onto microtiter plates. Following blocking with 3% BSA, appropriate wells were incubated with the indicated concentrations of recombinant RECQ1 (0–50 nM) for 1 h at 30°C. Parallel wells contained DNaseI (100 U/ml) or EtBr (50 µg/ml) in the binding step to test for DNA-mediated protein interaction. Following washing, Ku70/80-bound RECQ1 was detected by ELISA using anti-RECQ1 antibody. The values represent the mean of three independent experiments performed in duplicate with SD indicated by error bars. *C.* GST alone or GST-RECQ1 fragments (as indicated) bound to glutathione beads were incubated overnight at 4°C with HeLa extract (500 µg) that was either untreated or pre-treated with benzonase. After extensive washings, the bound Ku70/80 was eluted with SDS sample buffer and analyzed by Western blot using anti-Ku70 and anti-Ku80 antibodies (right). Coomassie staining of the eluted proteins was done to test expression of various GST-fusion fragments of RECQ1 (left). GST-RECQ1 proteins are marked by asterisk. Marker, protein molecular weight marker.

To investigate if the interaction between RECQ1 and Ku proteins is direct, we conducted GST-pull-down assays using bacterially purified proteins. The Ku70/80 complex consists of 70 kDa and 86 kDa subunits, and the Ku70/80 complex binds double-stranded and single-stranded DNA ends with high affinity [9]. Bacterially expressed His-tagged Ku70, Ku80 were examined for binding to a GST-tagged full length human RECQ1 (649 amino acids) *in vitro* (Fig. 2A). GST-RECQ1 interacted directly and independently with Ku70 and Ku80. Moreover, deletion of the DNA-PKcs interaction domain (amino acid residues 565–732) of Ku80 did not abolish its association with RECQ1 (Fig. 2A), indicating that RECQ1 and DNA-PKcs likely bind to different domains of Ku80.

A direct interaction between RECQ1 and Ku70/80 was confirmed by ELISA using purified recombinant proteins (Fig. 2B). RECQ1 bound Ku70/80 in a protein concentration-dependent manner, and a very low OD_490_ signal was detected in control experiments where BSA was substituted for Ku70/80. Incubation with either EtBr or DNase I during the binding did not affect the interaction appreciably, indicating that the interaction between Ku70/80 and RECQ1 is not DNA-dependent.

In order to map the Ku70/80 interaction domain(s) within RECQ1, GST fusion proteins that encompass truncated versions of human RECQ1 were used. These fusion proteins were expressed in bacterial cells and GST pull-down experiments were performed using HeLa cell extracts followed by Western blot analysis (Fig. 2C). Cellular Ku70/80 efficiently bound full-length RECQ1 in a DNA-independent manner. A polypeptide fragment carrying the C-terminus of RECQ1 (amino acid residues 592–649) efficiently bound Ku70 and Ku80. Moreover, RQC domain (amino acid residues 418–592) and helicase domain (amino acid residues 63–418) displayed a weaker binding to Ku70. Interestingly, when the genomic DNA in HeLa extract was eliminated by nuclease (benzonase) digestion prior to pull-down, Ku70/80 was found to efficiently bind the helicase domain of RECQ1 in addition to C-terminal domain (Fig. 2C). This suggests that the DNA binding modulates RECQ1-Ku70/80 interaction. It is possible that the RECQ1 helicase domain is alternatively engaged with either DNA or Ku70/80, or other unknown interacting protein(s) in the HeLa cell extract may compete with Ku70/80 for binding to RECQ1 [32], [33]. Altogether, these results demonstrate that RECQ1 forms a stable complex with Ku70/80 in human cells and a direct protein-protein interaction between RECQ1-Ku70/80 is mediated via the RECQ1 C-terminal domain with some contribution from the helicase core domain that is modulated by the presence of DNA.

### RECQ1 Binds and Unwinds Ku-bound DNA Substrate

Ku70/80 binds tightly to the ends of double-stranded DNA in a variety of structures and display equal affinity for 5′-protruding, 3′-protruding, and blunt end DNA [34]. The RECQ1 protein possesses a robust ATP-dependent 3′ to 5′ helicase activity, and has been shown to unwind a diverse set of DNA substrates [17], [18], [19], [35]. The finding that the RECQ1 protein interacts with Ku70/80 prompted us to ask whether Ku70/80 affects RECQ1 helicase activity.

**Figure 3:**
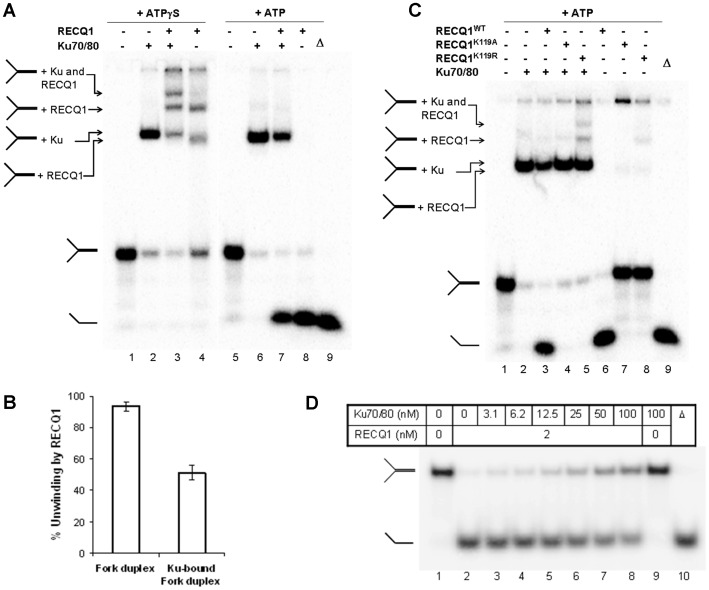
RECQ1 helicase binds and unwinds a Ku-bound forked duplex DNA substrate. *A.* RECQ1 binds and unwinds Ku-bound fork duplex. Ku70/80 (12.5 nM), RECQ1 (17.3 nM), or both were incubated with radiolabeled fork duplex (0.5 nM) in DNA binding buffer containing ATPγS or ATP (2 mm) as described in materials and methods. The protein-DNA complexes were then resolved on native 5% polyacrylamide gels. Radiolabeled bands were detected by PhosphorImager and a typical gel of resolved RECQ1/Ku70/80-fork duplex mixture is shown. Ku-DNA, RECQ1-DNA and RECQ1-Ku-DNA complexes are indicated based on their gel mobility shift. When binding reaction mixtures contained ATP, fork duplex, either alone or in complex with Ku70/80, was unwound by RECQ1 helicase resulting in the appearance of a faster migrating single stranded oligonucleotide as indicated. *B.* Quantitative comparison of RECQ1 unwinding of fork duplex or a Ku70/80-bound fork duplex assayed under DNA binding conditions in the presence of ATP (2 mM). The results shown are the average of at least three independent experiments, with SD indicated by error bars. *C.* Intrinsic ATPase activity of RECQ1 is essential for unwinding of Ku-bound fork duplex. Unwinding of Ku-bound fork duplex mediated by the RECQ1^K119A^, RECQ1^K119R^ or wild-type RECQ1^WT^ (each 17.3 nM) was assayed under DNA-binding conditions in the presence of ATP (2 mm). *D.* RECQ1 helicase activity is inhibited at several fold molar excess of Ku70/80. Helicase reactions containing fork duplex DNA substrate (0.5 nM) and the specified concentrations of Ku70/80 in the presence or absence of RECQ1 (2 nM) were incubated at 37°C for 15 min under standard helicase assay conditions as described in materials and methods. Phosphorimage of a typical gel is shown. Δ, heat-denatured DNA substrate control.

Purified recombinant proteins were used to perform *in vitro* assays in the presence of ATP or ATPγS (poorly hydrolyzable ATP analog) to measure DNA unwinding or binding, respectively. We utilized a forked DNA duplex with non-complementary 3′- and 5′-single strand DNA tails that is a preferred substrate for RECQ1 helicase [17] and model structural intermediates of DNA replication, repair, and recombination. Recombinant Ku70/80 protein (12.5 nM) bound efficiently and stably to the fork duplex in the presence of ATPγS or ATP, and the Ku70/80-bound fork duplex migrated slowly as a single shifted species (Fig. 3A, lane 2 and 6) as compared to the unbound fork in a 5% native gel (Fig. 3A, lane 1 and 5). Consistent with our previous report [17], RECQ1 (17.3 nM) efficiently bound the forked duplex substrate. In the presence of ATPγS, the RECQ1-bound fork duplex resulted in at least two retarded species (Fig. 3A, lane 4) possibly indicating DNA molecules bound by distinct RECQ1 protein assembly states or RECQ1 molecules bound to each single stranded arm of the forked duplex substrate [17], [36]. When both Ku70/80 and RECQ1 were incubated with fork duplex in the presence of ATPγS, we observed a super-shifted species that is distinct from those obtained with binding of the fork DNA with Ku70/80 or RECQ1 alone (Fig. 3A, lane 3), indicating that Ku70/80 and RECQ1 can together bind the fork DNA structure.

Since DNA helicases must load onto DNA substrates to initiate unwinding, we next examined whether RECQ1 can unwind a fork duplex that was pre-bound to Ku70/80. Ku70/80 binding to the DNA substrate was performed in the absence of ATP, followed by the subsequent addition of ATP and the RECQ1 helicase (Fig. 3A, lanes 5–8). In these reactions, Ku70/80 did not exhibit any detectable helicase activity but stably bound the fork duplex (Fig. 3A, lane 6). RECQ1 alone efficiently unwound the fork substrate and resulted in the appearance of a faster migrating species that co-migrated with the heat denatured DNA substrate (Fig. 3A, lane 8 and 9). Importantly, RECQ1 was also able to unwind Ku70/80-bound fork duplex (Fig. 3A, lane 7). Approximately, 51% of the Ku-bound fork duplex was unwound by RECQ1 (17.3 nM) as compared to 93% naked fork duplex substrate unwound by RECQ1 under identical reaction conditions (Fig. 3B).

To confirm that the observed unwinding of the fork duplex is strictly due to RECQ1 helicase activity, we utilized two independent and previously characterized ATPase/helicase dead mutants of RECQ1 [17]. In presence of ATP, wild-type RECQ1 effectively unwound the fork duplex whereas no detectable helicase activity was obtained by RECQ1^K119A^ or RECQ1K^119R^ mutant (Fig. 3C, lanes 6–8). Moreover, the helicase-dead RECQ1 failed to unwind the Ku70/80-bound fork duplex which was unwound by the wild-type RECQ1 (Fig. 3C, lanes 2–5). The RECQ1^K119R^ mutant is deficient in ATP hydrolysis but retains the ability to bind ATP and DNA [36]. Reactions containing both Ku70/80 and the RECQ1^K119R^ resulted in a distinct slow migrating species indicative of co-binding of the two proteins to the fork duplex (Fig. 3C, lane 5).

As shown in Fig. 3D, inhibition of RECQ1 helicase was only seen in the presence of several fold molar excess of Ku70/80 in unwinding reactions. Under optimal helicase reaction conditions, RECQ1 (2 nM) alone unwound approximately 93% of the fork duplex whereas 90% and 85% fork unwinding was displayed by RECQ1 in the presence of 6.2 nM and 12.5 nM Ku70/80, respectively (Fig. 3D, lanes 2–5). Approximately 54% of the fork duplex was unwound by RECQ1 (2 nM) in the presence of 100 nM Ku70/80 whereas no unwinding was detected for Ku70/80 alone in the given reaction conditions (Fig. 3D, lanes 8–9). Altogether, these results demonstrate that RECQ1 and Ku can bind a fork duplex and RECQ1 can unwind the Ku-bound forked duplex DNA substrate in a manner that is dependent on RECQ1 ATPase activity.

### RECQ1 and Ku70/80 co-bind Linear DNA

The interaction of RECQ1 with Ku70/80 led us to investigate whether RECQ1 could also interact with Ku when bound to a DNA substrate relevant to NHEJ. Although we have previously reported that RECQ1 binds but does not unwind a 44-bp blunt duplex [17], its ability to bind linearized double-stranded plasmid DNA has not been investigated. We employed EMSA to examine the DNA binding properties of RECQ1 and to test the interaction between the two proteins for DNA ends.

**Figure 4:**
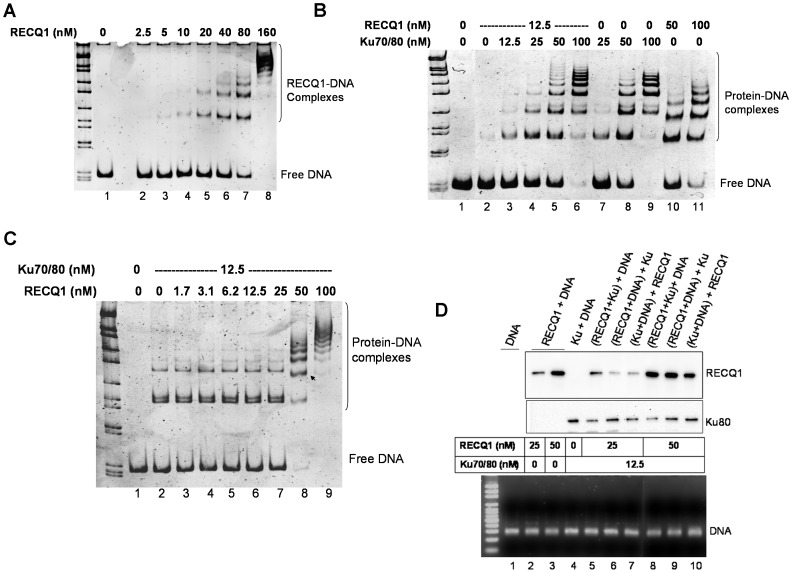
RECQ1 and Ku70/80 co-bind a linear blunt duplex DNA. *A.* RECQ1 binds a 322 bp blunt duplex fragment derived from pUC19 plasmid DNA. An electrophoretic mobility shift assay (EMSA) was performed to examine the ability of increasing concentration of purified RECQ1 to bind linearized plasmid DNA (30 ng) under DNA binding conditions as described in materials and methods. Protein-DNA complexes were resolved by native 6% polyacrylamide gels and detected by staining with SYBR Gold and a typical inverted image is shown. *B.* RECQ1 facilitates the formation of higher order DNA complex with Ku70/80. EMSA was performed to examine the ability of increasing concentration of Ku70/80 (0–100 nM) to interact with RECQ1 (12.5 nM) when bound to linear plasmid DNA (30 ng). Both RECQ1 and Ku70/80 bind to blunt duplex resulting in a series of progressively retarded bands, but the mobility of RECQ1-DNA and Ku70/80-DNA complex is distinct. Additional slow migrating bands were observed in Ku70/80 (25, 50 and 100 nM) in the presence of RECQ1 (12.5 nM) (lanes 4–6 and 7–9). *C.* Molar excess of RECQ1 may compete with Ku70/80 for DNA binding. EMSA was performed to examine the ability of increasing concentration of RECQ1 (0–100 nM) to interact with Ku70/80 (12.5 nM) when bound to linearized plasmid DNA (30 ng) (lane 7 and 8). Arrow indicates the change in band shift pattern of DNA-protein complexes at 50 nM RECQ1. *D.* RECQ1 and Ku70/80 are co-bound to linear blunt duplex. EMSA reactions were performed using biotinylated DNA probe and the indicated concentration of RECQ1, Ku70/80 or both. The DNA probe was either exposed to a mixture of RECQ1 and Ku70/80, or pre-incubated with one protein followed by addition of the second protein as indicated by parentheses. The DNA-protein complexes were pulled-down on streptavidin magnetic beads and DNA-bound RECQ1 and Ku70/80 were analyzed by Western blotting. Comparable amount of DNA was pull-down in all reactions as shown by agarose gel analyses (bottom). Lane 1 represents biotinylated DNA bound to the streptavidin beads in the absence of RECQ1 or Ku70/80. DNA size marker is shown in leftmost lane of each gel.

Purified recombinant RECQ1 and/or Ku70/80 were incubated with linearized double-stranded DNA, resolved by non-denaturing PAGE and stained with SYBR Gold to detect DNA-protein complexes. RECQ1 protein bound to a blunt ended 322 bp duplex in a protein concentration dependent manner giving rise to multiple RECQ1-DNA complexes of distinct mobility in 5% native gel (Fig. 4A). Ku70/80 also bound the linear DNA yielding a series of retarded bands representing Ku70/80-DNA complexes of increasing molecular mass (Fig. S2A); the observed pattern is consistent with earlier report that one molecule of Ku occupy/bind roughly 35 bp DNA like beads on string [37]. A higher concentration of RECQ1 (160 nM) was required to shift complete DNA as compared to Ku (100 nM) (Fig. 4A and B).

We next examined the DNA binding by RECQ1 (12.5 nM) in the presence of increasing concentration of Ku70/80 (0–100 nM) (Fig. 4B). The DNA-protein complexes of RECQ1 or Ku70/80 migrated with slightly differential mobility in native gel allowing us to compare the DNA binding in presence of both proteins (Fig. 4B, lanes 7–9 versus lanes 10–11). RECQ1 (12.5 nM) bound to the substrate DNA minimally to yield a weak but detectable complex (Fig. 4B, lane 2) and did not interfere with the Ku70/80-DNA binding as detected by the EMSA (Fig. 4B, lanes 3–6). Importantly, the presence of RECQ1 enhanced the appearance of slow migrating (higher order) complexes of Ku70/80 with DNA (Fig. 4B, lanes 4–6 versus lanes 7–9).

When a fixed concentration of Ku70/80 (12.5 nM) was pre-incubated with DNA and increasing amount of RECQ1(1.5–100 nM) was added, the band shifts maintained the Ku70/80 pattern until RECQ1 concentration reached ∼4 fold excess over Ku70/80 (Fig. 4C). Ku70/80-bound to the substrate DNA was observed as shifted gel bands and the banding pattern of DNA-protein complexes did not alter by the presence of up to 25 nM RECQ1 in the Ku70/80-DNA binding reactions (Fig. 4C, lanes 1–7). However, higher concentration of RECQ1 in the binding mixtures yielded a banding pattern that was distinct from RECQ1 or Ku70/80 alone at the given protein concentration (Fig.4C, lanes 8–9; Fig. 4A and 4B). Similarly, the presence of RECQ1 (160 nM) in Ku70/80 (100 nM)-DNA binding mixture resulted in a banding pattern that is distinct from the DNA-protein complexes generated from RECQ1 or Ku70/80 alone (Fig. S2B). The bands containing both Ku70/80 and RECQ1 displayed retarded mobility compared to those produced by either protein alone, suggesting that RECQ1 interacts with DNA-bound Ku70/80 and modulates its recruitment to DNA ends.

To confirm that both RECQ1 and Ku70/80 were indeed bound to the linearized 322 bp DNA fragment, and to test the effect of RECQ1 on DNA binding by Ku70/80, we used biotinylated DNA probe in binding mixtures containing recombinant RECQ1, Ku70/80 or both, and pulled down the DNA-protein complexes using streptavidin magnetic beads to analyze for DNA-bound proteins by Western blotting (Fig. 4D). To test the effect of order of addition in these experiments, DNA was either exposed to a mixture of Ku70/80 and RECQ1, or incubated with RECQ1 prior to addition of Ku70/80 and vice versa. In control experiments without DNA or with non-biotinylated DNA neither Ku70/80 nor RECQ1 co-precipitated with the streptavidin beads (not shown). RECQ1 or Ku70/80 was successfully bound to the biotinylated DNA probe (Fig. 4D, lanes 2–4) and both proteins were bound to the DNA when both RECQ1 and Ku70/80 were present in the reaction (Fig. 4D, lanes 5–10). Reduced Ku70/80 binding was observed when DNA was exposed to a mixture of RECQ1 (25 or 50 nM) and Ku70/80 (12.5 nM) as compared to Ku70/80 (12.5 nM) alone, whereas no such effect was seen for RECQ1 binding to DNA (Fig. 4D, lane 4 versus lanes 5 and 8). When the DNA was pre-incubated with RECQ1 (25 or 50 nM) followed by the addition of Ku70/80 (12.5 nM), the signal for DNA-bound Ku70/80 was found to be comparable to the Ku70/80 alone (Fig. 4D, lane 4 versus lanes 6 and 9); however, the signal for DNA-bound RECQ1 was reduced in these reactions as compared to RECQ1 alone or a mixture of RECQ1 and Ku70/80 (Fig. 4D, lanes 2, 3, 5 versus lanes 6, 8, 9). Similarly reduced DNA-bound RECQ1 was observed when RECQ1 was added to the Ku70/80-bound DNA (Fig. 4D, lanes 2, 3, 7 versus lanes 6, 8, 10) whereas the Ku70/80 binding to DNA was relatively unaffected (Fig. 4D, lanes 4, 7 versus lanes 6, 8, 10). Similar trend was observed in reactions containing excess Ku70/80 (160 nM) over RECQ1 (80 nM) (Fig. S3). Overall, in reactions where DNA was sequentially bound to RECQ1 and Ku70/80, RECQ1 promoted Ku70/80 binding to DNA regardless of the order of addition. In contrast, when DNA was exposed to a mixture of RECQ1 and Ku70/80, RECQ1 always bound to DNA more avidly whereas Ku lost it’s DNA-binding efficiency substantially, which led to predominant RECQ1 DNA occupancy. These results indicate that RECQ1 and Ku70/80 bind the DNA together but exhibit a competition for DNA binding.

### RECQ1 Modulates DNA End-joining

**Figure 5:**
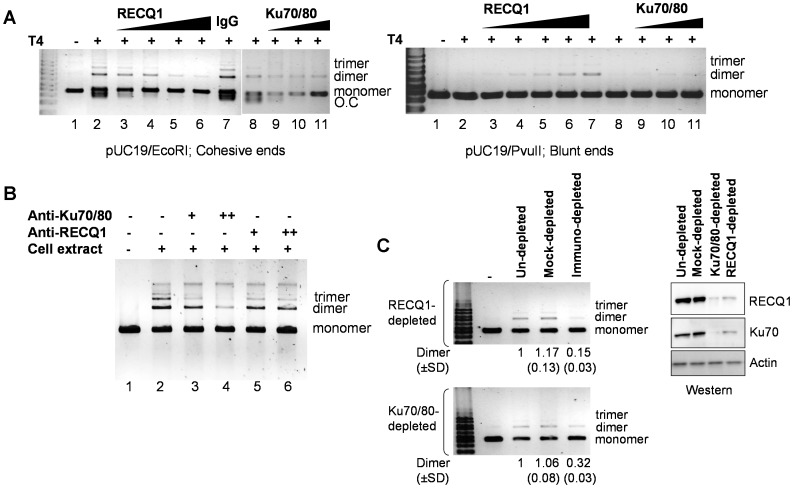
RECQ1 modulates DNA end-joining. *A.* RECQ1 affects DNA end-joining by T4 ligase. Comparison of ligation products of 5′-cohesive (left panel) or blunt (right panel) ended linear DNA after incubation with T4 ligase alone, or after pre-incubation with the increasing amount of RECQ1 (0–260 ng) or Ku70/80 (0–400 ng) as described in materials and methods. IgG (1µg) was included as unrelated protein in a control reaction. Linear substrate DNA is indicated as monomer, and the end-joined products corresponding to dimer and trimer are indicated. O.C., open circular. DNA size marker is shown in leftmost lane. *B.* RECQ1 antibody interferes with cell free extract mediated end-joining *in vitro*. The effect of antibodies against human RECQ1 (1.5 and 3 µg) or Ku70/80 (1 and 2 µg) was tested in DNA end-joining reactions assembled with 5 µg HeLa cell free extract. Antibodies at the indicated amounts were incubated with cell free extract for 10 min at room temperature in NHEJ buffer without DNA and ATP. Subsequently, EcoRI-linearized pUC19 DNA and ATP were added to start the reaction followed by 2 h incubation at room temperature. Reaction products were purified and analyzed by SYBR Gold staining after resolution on agarose gel. Linear substrate DNA is indicated as monomer, and the end-joined products corresponding to dimer and trimer are indicated. *C.* Immunodepletion of RECQ1 or Ku70/80 from cell extracts results in similarly reduced end-joining. DNA end joining reactions were performed using HeLa extract depleted either with anti-RECQ1 polyclonal antibody (upper panel) or with the anti-Ku70/80 monoclonal antibody (lower panel) as described. Mock-depleted extracts used preimmune rabbit or mouse IgG as control for RECQ1 or Ku70/80 depletion, respectively. Linear substrate DNA is indicated as monomer, and the end-joined products corresponding to dimer and trimer are indicated. Inverted image of a typical gel is shown. ImageJ was used to quantitate dimer product in each case, and the average from at least three independent experiments are indicated including SD. Western blot of control un-depleted, mock-depleted and RECQ1 or Ku70/80 depleted extracts used in the end-joining reaction is shown along with a loading control (actin) (right).

The interaction of RECQ1 with Ku70/80, and their mutual binding to DNA suggested that RECQ1 might help to bring DNA ends in close proximity to facilitate DSB repair by NHEJ. We investigated the influence of RECQ1 on ligation of DSBs by T4 DNA ligase *in vitro*. RECQ1, however, did not promote the end-joining of EcoRI-linearized pUC19 plasmid DNA with 5′-cohesive ends by T4 DNA ligase (Fig. 5A, left panel). In a standard ligation reaction, T4 ligase promoted both intra- and inter-molecular joining of the cohesive DNA ends; the end-joined products contained higher oligomeric forms representing linear multimers and open circular plasmid (Fig. 5A, lane 2). The presence of RECQ1 in T4 ligase reaction resulted in inhibition of ligation in a RECQ1 protein concentration dependent manner (Fig. 5A, lane 2 versus lanes 3–6). In comparison, the plasmid DNA was efficiently ligated in the presence of a non-related control protein (IgG) (Fig. 5A, lanes 2 versus 7). The failure to observe ligation product in presence of RECQ1 suggests that RECQ1 binding to the DNA ends prevents access to T4 DNA ligase. When blunt-ended linear DNA was used as the substrate, the activity of T4 DNA ligase was low, producing a small amount of dimeric forms of the linear DNA substrate (Figure 5A, lane 2, right panel). The addition of RECQ1 had a weak stimulatory effect (Figure 5A, lanes 3–6, right panel). Similar results were obtained with Ku70/80 (Figure 5A, lanes 9–11, both panels). This is consistent with previous report where the ligation efficiency with T4 DNA ligase was stimulated minimally in the presence of Ku70/80, and addition of Ku protein actually inhibited the activity of T4 ligases under conditions that promote efficient ligation, such as cohesive DNA ends [38]. It has been suggested that the structure of Ku-bridged DNA ends may restrict prokaryotic ligases but permit eukaryotic ligases to perform ligation [38].

To explore a role of RECQ1 in NHEJ, we utilized an established *in vitro* assay to measure end-joining using HeLa cell extract and EcoRI-linearized pUC19 DNA as substrate [30], [39]. Cell free extract mediated plasmid end-joining was shown to be inhibited by the presence of Ku70/80 antibody in similar *in vitro* assay [40]; therefore we first examined the effect of adding RECQ1 or Ku70/80 antibodies to the NHEJ reaction. Resolution of the end-joining reaction products by agarose gel electophoresis displayed a series of bands representing multimerization of linear DNA by ligation of the cohesive ends (Fig. 5B, lane 2); higher oligomeric forms representing dimer, trimer and tetramer of linear substrate DNA were resolved as distinct bands. No ligation was obtained when heat denatured HeLa cell extract was used in reaction (data not shown). Addition of antibody against Ku70/80 inhibited the ligation in protein concentration dependent manner and consequently reduced multimerization of linear plasmid DNA was seen (Fig. 5B, lane 2 versus lanes 3–4). Addition of the same amounts of rabbit or mouse IgG did not influence end-joining (data not shown). Notably, the antibody directed against RECQ1 elicited a qualitatively similar response, reducing the formation of multimers, although to a lesser extent than Ku70/80 antibody, suggesting participation of RECQ1 in DNA end-joining in this *in vitro* assay (Fig. 5B, lane 2 versus lanes 5–6).

To obtain experimental evidence if RECQ1-containing complexes influence DNA end-joining, we immunodepleted the HeLa cell extracts by using specific antibodies directed against RECQ1 or Ku70/80 (Fig. 5C). Similar amount of rabbit or mouse IgG were used for mock-depletion in RECQ1 or Ku70/80 immunodepletion panels, respectively. Extract exposed to the beads without any antibody served as un-depleted control. Efficient depletion of RECQ1 or Ku70/80 was confirmed by Western analysis; a small but detectable amount of both RECQ1 and Ku70/80 remained in the extracts (Fig. 5C). Importantly, immunodepletion of RECQ1 from the HeLa cell extracts also depleted Ku70/80 and vice versa (Fig. 5B, right panel) further supporting their physical interaction *in vivo*. RECQ1 immunodepletion inhibited multimerization; only ∼15% of the dimer formation was observed in RECQ1-depleted extract as compared to un-depleted or mock-depleted extract (Fig. 5C, lane 4 versus 2–3, upper panel). Immunodepletion of Ku70/80 also inhibited end-joining and ∼32% dimer formation was obtained in Ku70/80-depleted extract as compared to un-depleted or mock-depleted extract (Fig. 5C, lane 4 versus 2–3, lower panel). These *in vitro* results suggest that RECQ1 in complex with Ku70/80 is involved in the NHEJ pathway of DSB repair.

### RECQ1-deficient Cells Promote End-joining but show Defective Ku-DNA Binding

**Figure 6:**
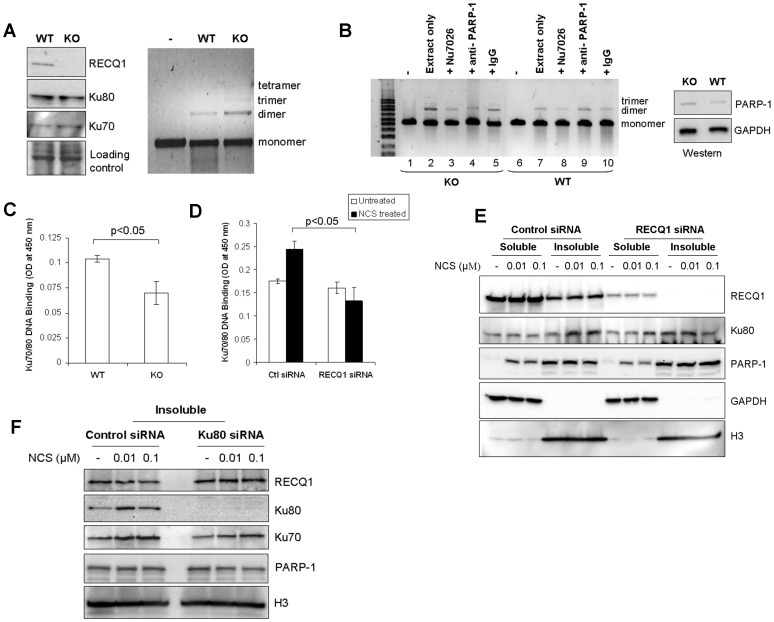
RECQ1-null cells promote end-joining but show reduced Ku-DNA binding activity. *A.* Level of *in vitro* end-joining activity is comparable in WT and RECQ1 KO MEFs. Cell free extracts prepared from non-transformed RECQ1 WT or KO MEFs were used in end-joining reaction containing EcoRI-linearized pUC19 DNA as substrate. Linear substrate DNA is indicated as monomer, and the end-joined products corresponding to dimer, trimer and tetramer are indicated (right panel). Western blot showed no detectable difference in Ku protein level in WT and KO cell extracts (left panel). GAPDH serves as loading control. *B.* Presence of PARP-1 antibody interferes with RECQ1 KO cell free extract mediated end-joining. In addition to standard reactions, *in vitro* end-joining reactions were performed with WT or KO cell extracts in the presence of a DNA-PKcs inhibitor Nu7026 (1.2 µM) or a specific anti-PARP-1 antibody (3 µg). IgG (3 µg) was included as unrelated antibody in a control reaction. Linear substrate DNA is indicated as monomer, and the end-joined products corresponding to dimer and trimer are indicated. Western blot showed no detectable difference in PARP-1 protein level in WT and KO cell extracts (left panel). GAPDH serves as loading control. *C.* Ku70/80 DNA binding assay performed by using Active Motif kit shows diminished DNA binding in RECQ1 KO extract as compared to WT extract (p<0.05). The results shown are the average of at least three independent experiments, with SD indicated by error bars. *D.* DNA binding analysis for Ku70/80 in extracts prepared from control or RECQ1-depleted HeLa cells. Nuclear extracts prepared from control or RECQ1 siRNA transfected cells either untreated or treated with NCS (0.01 µM, 3 h) were used to perform Ku70/80 DNA binding assay (Active Motif). Following NCS treatment, RECQ1 siRNA transfected cells showed significantly reduced Ku70/80 DNA binding activity as compared to control siRNA transfected cells (p<0.05). The results shown are average of at least three independent experiments, with SD indicated by error bars. *E.* RECQ1-depleted HeLa cells show reduced chromatin bound Ku following NCS treatment. Ku80 and PARP-1 was detected by Western blot in the soluble and insoluble fractions prepared from control or RECQ1 siRNA transfected cells that were either untreated or treated with indicated dose of NCS for 3 h. GAPDH serves as cytoplasmic marker and H3 serves as marker for chromatin enriched fractions. *F.* RECQ1 protein in the chromatin enriched fraction of control or Ku80-depleted cells. Cells were untreated or treated with indicated dose of NCS for 3 h, followed by biochemical fractionation and the chromatin containing insoluble fractions were examined by Western blotting. H3 serves as marker for chromatin enriched fractions.

We next compared the status of NHEJ in the extracts prepared from RECQ1-deficient or proficient cells. Cell free extracts were prepared from the WT or RECQ1 KO MEFs. Western analyses demonstrated no significant difference in the protein level of Ku70/80 in WT and KO cell extracts (Fig. 6A). Equal protein amounts of the WT or KO extracts were incubated with EcoRI-linearized plasmid DNA in NHEJ reactions. Resolution of the end-joined products on agarose gel revealed formation of dimer of linear substrate DNA by both WT and KO MEFs (Fig. 6A); however, a greater signal for dimer product was consistently obtained by KO extract as compared to WT under same NHEJ reaction condition. Interestingly, end-joined products from the KO MEFs also contained trimer and tetramer of linear substrate DNA, whereas these higher multimers were not visible in WT MEFs reactions (Fig. 6A). Thus, extracts prepared from RECQ1 KO MEFs promote end-joining, and appear to be slightly more efficient than extracts from WT MEFs in NHEJ *in vitro*.

Substantial DNA end-joining has been reported in cells deficient in Ku70 or Ku80 indicating the presence of an alternative pathway of end-joining as backup to the classical pathway of NHEJ [41], [42]. Thus, NHEJ consists of at least two sub pathways: the main Ku70/80/DNA-PK dependent “classic” NHEJ pathway and an “alternative” NHEJ pathway that requires PARP-1 and other factors [43]. We used a selective DNA-PK inhibitor or antibody against PARP-1 to determine whether the end-joining in WT and KO MEF extracts is dependent on DNA-PK and/or PARP-1. Presence of Nu7026 inhibited the formation of multimers in both KO and WT extracts, although to a different extent (Fig. 6B, lanes 2, 3, 7 and 8). As compared to the dimer product formed in control end-joining reactions, only 19% (±4 SD) dimer formation was observed in the presence of Nu7026 in WT reactions whereas 32% (±3 SD) dimer and residual trimer of linear substrate DNA were still present in KO reactions performed in the presence of Nu7026. Presence of a specific PARP-1 antibody in NHEJ reaction resulted in inhibition of end-joining by KO extract and only 22% (±4 SD) dimer was obtained (Fig. 6B, lane 2 versus 4); no significant effect was observed in end-joining by WT extracts and more than 95% (±2 SD) dimer formation was observed in WT reactions performed in the presence of PARP-1 antibody (Fig. 6B, lane 3 versus 9). Thus, PARP-1 antibody resulted in greater interference in end-joining in RECQ1 KO cell extract as compared to WT extracts.

Since the DNA-binding activity of the Ku70/80 is critical for classical NHEJ, we utilized a quantitative assay to determine the DNA binding activity of Ku70/80 in RECQ1-deficient and proficient cells. Extracts prepared from the KO MEFs displayed significantly reduced Ku70/80 DNA binding activity as compared to the extracts from the WT MEFs (Fig. 6C); the binding of Ku70/80 to DNA was reduced to approximately 68% in RECQ1 KO MEFs as compared to WT MEFs. Acute depletion of RECQ1 by siRNA also resulted in reduced DNA binding of Ku70/80 in HeLa cells (Fig. 6D and 6E). Ku70/80 binding to DNA was reduced to about 91% and 54% in nuclear extracts prepared from RECQ1-depleted untreated or NCS-treated cells as compared to control siRNA transfected cells, respectively (Fig. 6D). As reported previously [31], NCS treatment enriched Ku70/80 in the insoluble fraction that also contained histones (Fig. 6E). The chromatin enriched fractions of RECQ1-depleted cells displayed reduced Ku80 as compared to control siRNA transfected cells following treatment with 0.1 µM NCS (Fig. 6E); quantification of Western signal demonstrated a 2.1 (±0.3 SD) fold greater signal for chromatin bound Ku80 in control siRNA transfected cells as compared to RECQ1-depleted cells (Fig. 6E). Furthermore, RECQ1 siRNA transfected HeLa cells displayed a 2.4 (±0.4 SD) fold greater signal for chromatin bound PARP-1 as compared to control siRNA transfected cells following treatment with 0.1 µM NCS (Fig. 6E). Examination of RECQ1 in chromatin enriched fractions of Ku80-depleted cells revealed a modest increase in RECQ1 (1.54 (±0.4 SD) fold) as compared to control cells following NCS treatment (0.1 µM) (Fig. 6F). These results suggest that RECQ1 plays a role in regulating the DNA binding activity of Ku70/80.

## Discussion

We have identified a reciprocal, DNA-independent interaction of RECQ1 and Ku70/80 in cell extracts. This interaction is mediated by the C-terminal domain of RECQ1, and RECQ1 and Ku70/80 can simultaneously bind linearized plasmid DNA *in vitro*. RECQ1 can unwind Ku-bound DNA duplex in a manner that is dependent on intrinsic RECQ1 ATPase activity. Finally, RECQ1 binds at DNA ends and modulates end-joining in cell free extracts. Collectively, these results demonstrate a physical and functional interaction between RECQ1 and Ku70/80, and suggest a role of RECQ1 in DSB repair via NHEJ. This newly identified role of RECQ1 is compatible with the demonstrated functions of RecQ homologs in DNA repair [44].

Interaction with Ku70/80 supports a putative role of RECQ1 in NHEJ. Our data indicate that RECQ1 binds to individual subunits of the Ku70/80 heterodimer. RECQ1 binding to Ku70/80 involves its poorly conserved C-terminus, and also helicase domain when DNA is absent. The observed alternative binding of the RECQ1 helicase domain with either Ku70/80 or DNA may be due to competition with other interacting proteins present in the cell extract or a DNA-induced structural change in proteins. Indeed, dynamic oligomerization of RECQ1 has been reported in the presence of DNA [17], [36]. In the context of NHEJ, Ku70/80 first binds the DSB, followed by recruitment of DNA-PKcs which leads to stimulation of DNA-PKcs protein kinase activity, autophosphorylation, and dissociation of DNA-PKcs [9]. Endogenous RECQ1 is phosphorylated in response to DNA damage [25] but whether RECQ1 is a target of phosphorylation by DNA-PK is not known. RECQ1 bound a truncated Ku80 protein lacking the extreme C-terminus region important for interaction with DNA-PKcs suggesting that the Ku70/80 heterodimer is capable of binding to DNA-PKcs and RECQ1 concurrently. Overall results presented here show that the physical interaction of RECQ1 with Ku70/80 is independent of DNA-PKcs.

Upon binding to a free DNA end, Ku70/80 can translocate internally on a DNA fragment leaving the ends free to bind additional protein molecules; thus multiple Ku molecules can bind to the same DNA molecule like beads on a string to give multimeric Ku-DNA complexes, [34], [37], [45]. We note a remarkable similarity in the band shift pattern of the DNA-protein complexes of RECQ1 and Ku70/80 with a 322 bp blunt duplex DNA. Appearance of a “ladder” of progressively retarded bands in a RECQ1 concentration dependent manner suggests formation of regular multimeric DNA-protein complexes. Further studies are needed to elucidate the DNA binding properties of RECQ1, but the oligomeric ring-like structure of RECQ1 may allow it to thread on double-stranded DNA [36]. Our studies suggest that the Ku heterodimer and RECQ1 bind independently to double-stranded DNA. Ku70/80 is reported to be in dynamic equilibrium between the DNA bound and free states [46]. In principle, free DNA ends might lose juxtaposition needed for joining unless some other protein bridge keeps them together; it is conceivable that RECQ1 binds the DNA ends and facilitates this step. Effective inhibition of T4 ligase mediated joining of linear DNA molecules by RECQ1 indicated the presence of RECQ1 protein on the DNA ends providing another functional correlate with Ku70/80 [38].

We provide biochemical evidence for the RECQ1-Ku-DNA complex *in vitro*. Biotinylated DNA pull-down experiments revealed that the association of RECQ1 and Ku70/80 with linear DNA depends upon their relative abundance and order of addition. Our results indicate that RECQ1 could compete with Ku for the DNA ends when both proteins are available to the linear DNA. This characteristic may be critical for the repair of lesions that arise from stalled or broken replication forks since Ku binds to such structures and inhibits access of other repair factors to the DNA [47]. *In vivo*, the high affinity binding of Ku to DNA ends, necessary for tethering DNA ends in close proximity for repair by NHEJ, limits DNA end resection which is a major prerequisite for HR [48]. Recent studies support a model in which Ku binds DNA ends first and then is subsequently released from the DNA through the DNA end processing activities provided by the MRN complex, CtIP and Exo-1 [48], [49]. Nuclease activities of the MRN complex and CtIP are required to initiate DSB resection [50], [51], [52], and further resection involves participation of Exo-1 alone or a RecQ helicase (WRN, BLM, yeast Sgs1) in conjunction with either Exo-1 or Dna2 [53], [54], [55], [56], [57], [58]. Ku inhibits Exo-1-mediated DSB resection in human cells [59] and the addition of Ku70/80 blocked Exo-1-mediated DNA end resection of the forked duplex substrate *in vitro* [60]. Notably, RECQ1 physically interacts with Exo-1 and stimulates its 5′-3′ exonuclease activity [29]. Our current finding that RECQ1 can bind and unwind a Ku-bound fork duplex substrate relatively efficiently suggests that RECQ1 may enable Exo-1 to overcome Ku inhibition. To our knowledge, this is the first demonstration that a human motor ATPase (RECQ1) can displace Ku protein complex bound to DNA, which likely has implications for how Ku governs DNA repair pathway choice.

Depletion of RECQ1 from cell extracts resulted in co-depletion of Ku and reduced end-joining of plasmid DNA *in vitro*. Thus, although dispensable for NHEJ in a reconstituted *in vitro* system [61], our results provide initial evidence for the involvement of RECQ1 in NHEJ as a component of a Ku-containing multi-protein complex. Contrary to what we predicted, extracts prepared from the RECQ1-null cells displayed wild-type level of end-joining. However, RECQ1-deficient cells show reduced DNA binding activity of Ku, and end-joining in the absence of RECQ1 is likely promoted by an alternative pathway since the extracts prepared from RECQ1 null cells show greater reliance on PARP-1 for joining of linear plasmid DNA ends *in vitro*. This is reminiscent of our recent observation that RECQ1-deficient cells hyperactivate PARP-1 in response to DNA strand breaks induced by reactive oxygen species [26]. Cell lines deficient in Ku70 or Ku80 carry out proficient end-joining due to hyperactive alternate end-joining [42] and a requirement for PARP-1 has been shown for alternate end-joining in the absence of Ku [62]. Ku inhibits alternate end-joining in mammalian cells [42] and competes with PARP1 for DNA ends *in vitro* [63], supporting the notion that Ku generally gets to the ends of a DSB faster and with greater affinity than PARP-1 (and other DNA-binding factors) and blocks their access to promote DSB repair via classical end-joining [42]. RECQ1 is present with PARP-1 and Ku80 in a multi-protein complex of APLF (APTX -PNK-Like Factor) which is implicated in recruitment of NHEJ factors at DSBs [32], [64], [65], [66]. In addition to interacting with each other, RECQ1 and Ku are capable of interacting with PARP-1 indicating a functional interplay in the repair of DNA strand breaks (Fig. S4) [26], [67]. Although a precise role of RECQ1 in maintaining genomic stability remains to be characterized, we propose that the DNA binding ability of RECQ1 and its protein interactions play an important role in the modulation of DSB repair by NHEJ.

Similar to RECQ1, WRN is another human RecQ protein that interacts with both Ku70/80 [68] and PARP-1 [69]. Ku70/80 stimulates WRN exonuclease activity [68], [70], [71], whereas PARP-1 inhibits both the exonuclease and DNA helicase activities of WRN *in vitro* [72]. Furthermore, PARP-1 poly-ADP-ribosylates Ku70/80 *in vitro*, resulting in the loss of their DNA binding activities and the decrease of Ku70/80-mediated stimulation of WRN exonuclease activity [73]. Error-prone end-joining has been reported in Werner Syndrome patient cells [74]. However, unlike RECQ1, WRN-deficiency is characterized by reduced HR [75] and defective PARP-1 activation upon strand breaks [26]. Thus, RecQ helicases, likely in concert with their protein partners, contribute to varying outcomes of repair and disease. Recently, up-regulation of WRN was associated with increased activity of an alternative NHEJ repair pathway [76]. Alternative end-joining has been implicated in the generation of large deletions and other genomic rearrangements, including those found in human cancers [77]. RECQ1-deficiency leads to increased sensitivity to ionizing radiation, spontaneous chromosomal breakage, and frequent translocation events [24], [25]. Although the end-joining efficiency of RECQ1-null cells is not compromised, the fidelity of joining remains to be characterized. It is conceivable that the alternative end-joining contributes to the reported chromosomal instability in RECQ1-deficient cells [22], [24]. Cellular phenotypes of RECQ1-deficiency and its interaction with proteins involved in regulation of genetic recombination has suggested a role in HR but recent data do not support a prominent role of RECQ1 in DSB-induced HR [22], [26]. Results presented here associate RECQ1 with NHEJ and underscore the need to elucidate how the activities of human RecQ proteins participate in dedicated pathways or sub pathways of DSB repair for genome maintenance.

*Note added in proof:* While this work was under review, Vindigni and colleagues also reported Ku70/80 and PARP1 in complex with RECQ1 [78].

## Supporting Information

Figure S1 **Immunofluorescence staining of endogenous RECQ1 and Ku70/80 in DNA-PKcs deficient MO59J cells.** Cells grown on coverslips were either mock-treated or treated with NCS (100 ng/ml, 3 h). Cells were fixed and immunostained using a mouse monoclonal Ku70/80 antibody (1∶200) and a rabbit polyclonal RECQ1 antibody (1∶500). RECQ1 and Ku70/80 were visualized with Alexa Fluor 488- or Alexa Fluor 568-conjugated secondary antibodies, respectively, followed by microscopy.
(TIF)

Figure S2 **RECQ1 and Ku70/80 co-bind a linear blunt duplex DNA.** *A.* Ku70/80 binds a 322 bp blunt duplex fragment derived from pUC19 plasmid DNA. EMSA was performed to examine the ability of increasing concentration of purified Ku70/80 to bind linearized plasmid DNA (30 ng) under DNA binding conditions as described in materials and methods. DNA-protein complexes were resolved by native 6% polyacrylamide gels and detected by staining with SYBR Gold. *B.* Supershifting of linear blunt duplex DNA by RECQ1 and Ku70/80. EMSA was performed in the presence of Ku70/80 (100 nM), RECQ1 (160 nM) or both. As compared to Ku70/80 or RECQ1 alone, additional slow migrating bands of DNA-protein complex were observed when both Ku70/80 and RECQ1 were present.
(TIF)

Figure S3 **Co-binding of the substrate DNA with RECQ1 and Ku is modulated by the order of addition** ***in vitro*** **.** EMSA reactions were performed using biotinylated DNA probe and the indicated concentration of RECQ1, Ku70/80 or both. The DNA probe was either exposed to a mixture of RECQ1 and Ku70/80, or pre-incubated with one protein followed by addition of the second protein as indicated by parentheses. The DNA-protein complexes were pulled-down on streptavidin magnetic beads and DNA-bound RECQ1 and Ku70/80 were analyzed by Western blotting. Comparable amount of DNA was pull-down in all reactions as shown by agarose gel analyses (bottom). DNA size marker is shown in the first lane.
(TIF)

Figure S4 **Ku70/80 immunoprecipitate contains RECQ1 and PARP-1.** Ku70/80 was immunoprecipitated from HeLa nuclear extract with antibody specific for Ku70/80 or preimmune IgG as indicated. Eluted proteins in immunoprecipitate were analyzed by Western blotting and are indicated. Input represents 5% of total protein used in IP reactions.
(TIF)
