# Supplementary figures and images for: Human RECQ1 Interacts with Ku70/80 and Modulates DNA End-Joining of Double-Strand Breaks
Source: PLoS One. 2013 May 1;8(5):e62481. doi: 10.1371/journal.pone.0062481 (PMC3641083; doi:10.1371/journal.pone.0062481)

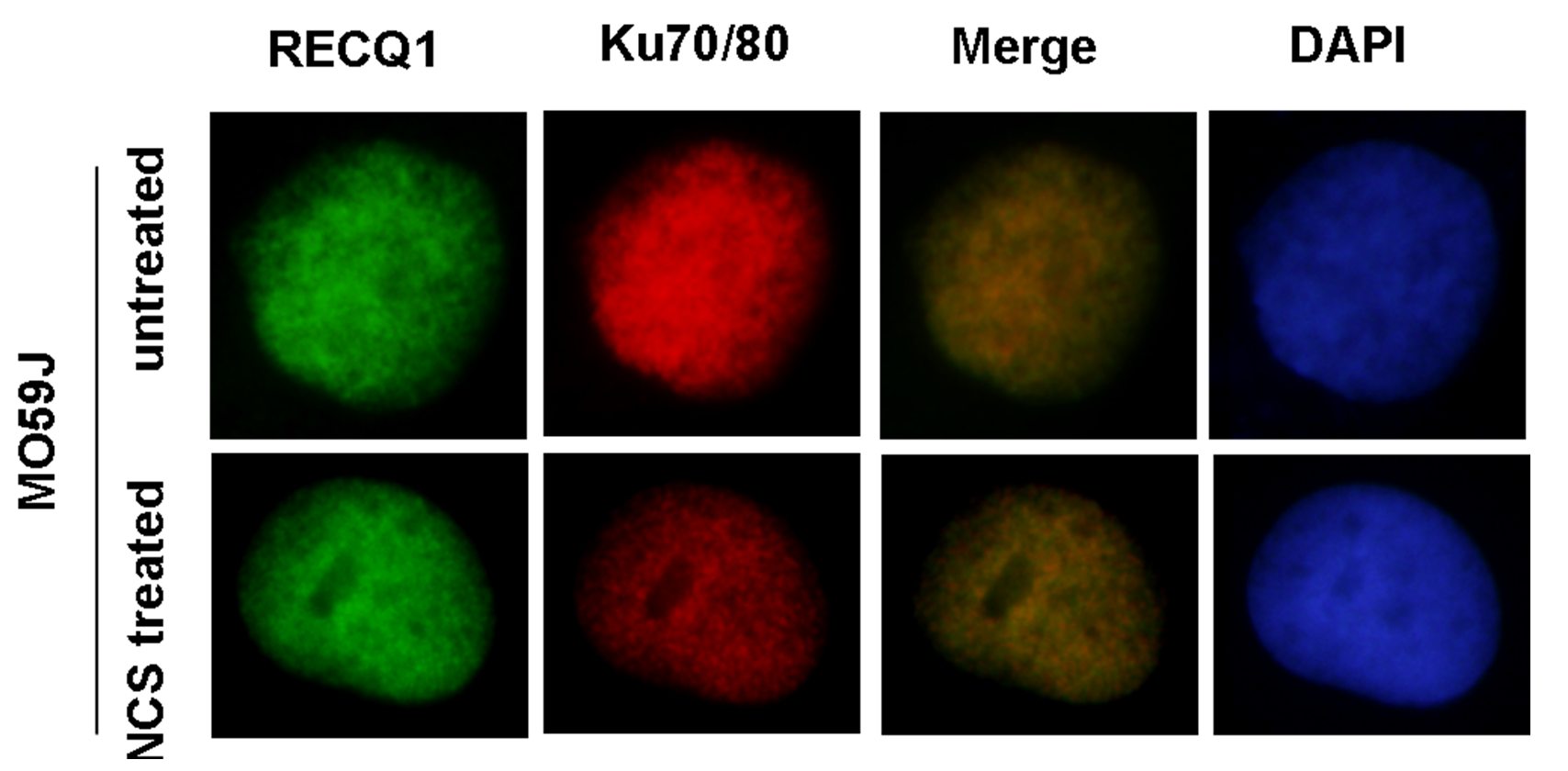

Supplement: Figure S1 — Immunofluorescence staining of endogenous RECQ1 and Ku70/80 in DNA-PKcs deficient MO59J cells. Cells grown on coverslips were either mock-treated or treated with NCS (100 ng/ml, 3 h). Cells were fixed and immunostained using a mouse monoclonal Ku70/80 antibody (1∶200) and a rabbit polyclonal RECQ1 antibody (1∶500). RECQ1 and Ku70/80 were visualized with Alexa Fluor 488- or Alexa Fluor 568-conjugated secondary antibodies, respectively, followed by microscopy. (TIF) [file pone.0062481.s001.tif]

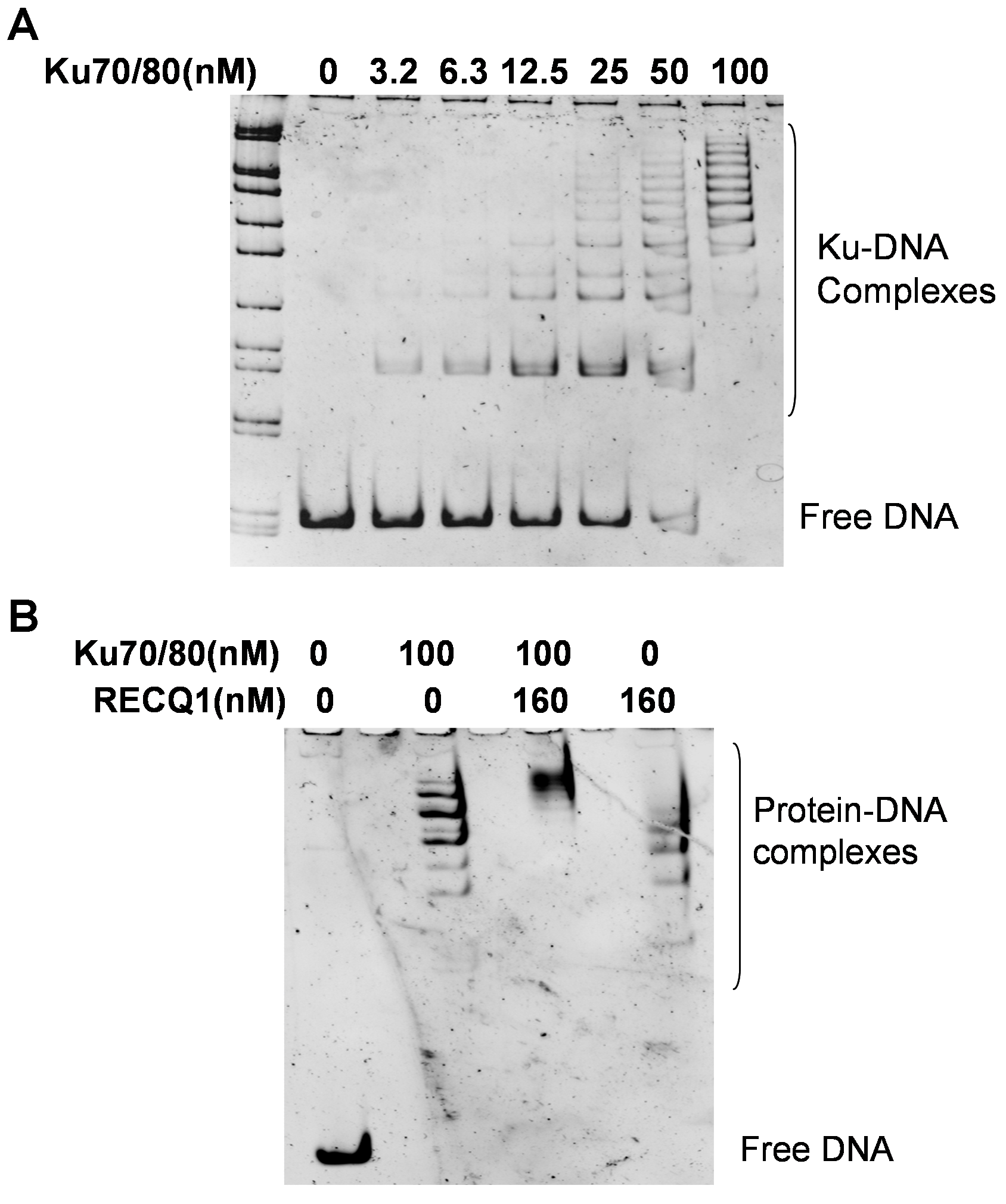

Supplement: Figure S2 — RECQ1 and Ku70/80 co-bind a linear blunt duplex DNA. A. Ku70/80 binds a 322 bp blunt duplex fragment derived from pUC19 plasmid DNA. EMSA was performed to examine the ability of increasing concentration of purified Ku70/80 to bind linearized plasmid DNA (30 ng) under DNA binding conditions as described in materials and methods. DNA-protein complexes were resolved by native 6% polyacrylamide gels and detected by staining with SYBR Gold. B. Supershifting of linear blunt duplex DNA by RECQ1 and Ku70/80. EMSA was performed in the presence of Ku70/80 (100 nM), RECQ1 (160 nM) or both. As compared to Ku70/80 or RECQ1 alone, additional slow migrating bands of DNA-protein complex were observed when both Ku70/80 and RECQ1 were present. (TIF) [file pone.0062481.s002.tif]

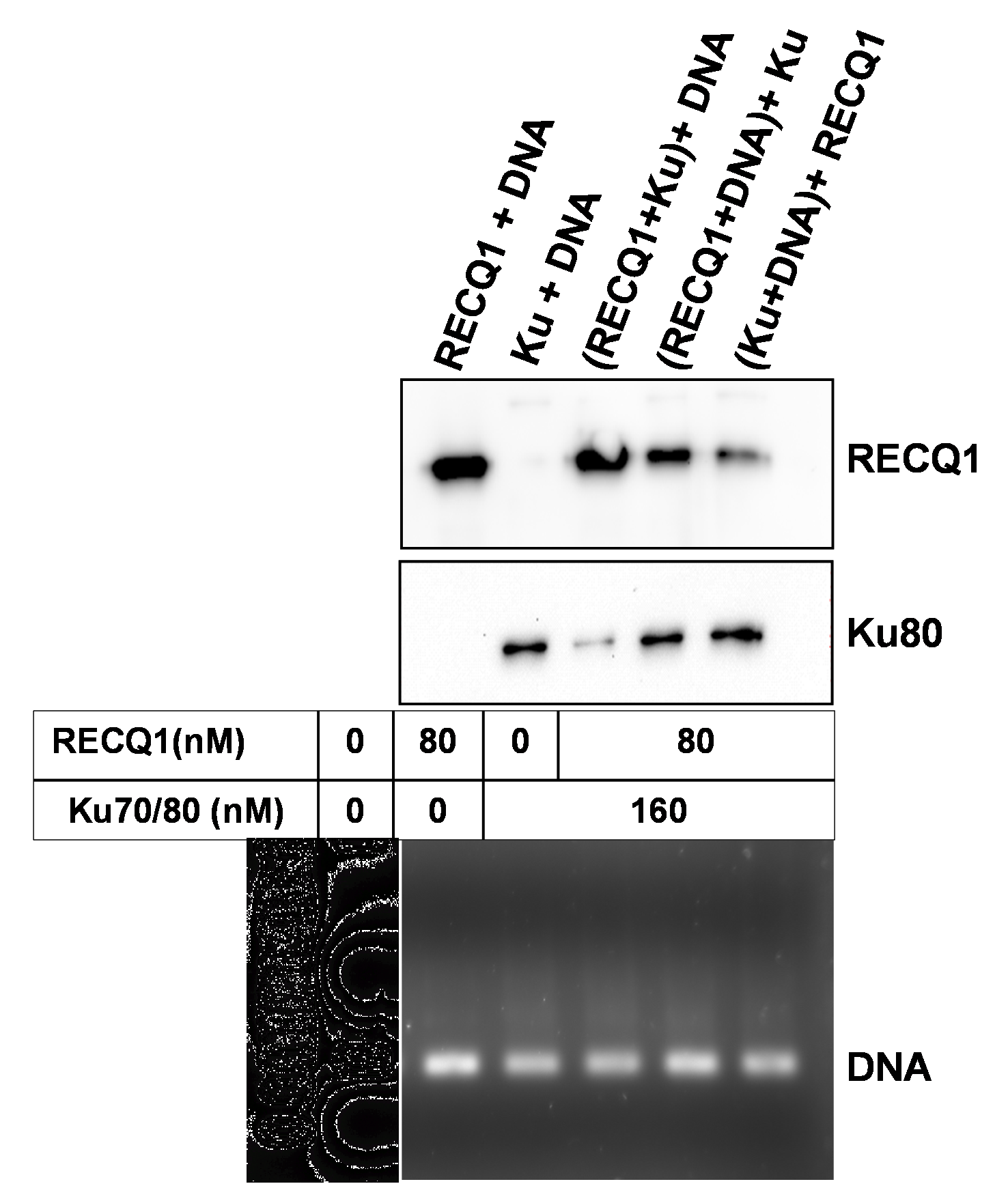

Supplement: Figure S3 — Co-binding of the substrate DNA with RECQ1 and Ku is modulated by the order of addition in vitro . EMSA reactions were performed using biotinylated DNA probe and the indicated concentration of RECQ1, Ku70/80 or both. The DNA probe was either exposed to a mixture of RECQ1 and Ku70/80, or pre-incubated with one protein followed by addition of the second protein as indicated by parentheses. The DNA-protein complexes were pulled-down on streptavidin magnetic beads and DNA-bound RECQ1 and Ku70/80 were analyzed by Western blotting. Comparable amount of DNA was pull-down in all reactions as shown by agarose gel analyses (bottom). DNA size marker is shown in the first lane. (TIF) [file pone.0062481.s003.tif]
